# Interaction of client—the scaffold on which FeS clusters are build—with J-domain protein Hsc20 and its evolving Hsp70 partners

**DOI:** 10.3389/fmolb.2022.1034453

**Published:** 2022-10-12

**Authors:** Jaroslaw Marszalek, Elizabeth A. Craig

**Affiliations:** ^1^ Intercollegiate Faculty of Biotechnology, University of Gdansk and Medical University of Gdansk, Gdansk, Poland; ^2^ Department of Biochemistry, University of Wisconsin—Madison, Madison, WI, United States

**Keywords:** molecular chaperones, FeS cluster biogenesis, Isu/IscU scaffold, Hsp70-client interaction cycle, protein interactions, protein evolution, post-duplication functional divergence

## Abstract

In cells molecular chaperone systems consisting of Hsp70 and its obligatory J-domain protein (JDP) co-chaperones transiently interact with a myriad of client proteins—with JDPs typically recruiting their partner Hsp70 to interact with particular clients. The fundamentals of this cyclical interactions between JDP/Hsp70 systems and clients are well established. Much less is known about other aspects of JDP/Hsp70 system function, including how such systems evolved over time. Here we discuss the JDP/Hsp70 system involved in the biogenesis of iron-sulfur (FeS) clusters. Interaction between the client protein, the scaffold on which clusters are built, and its specialized JDP Hsc20 has stayed constant. However, the system’s Hsp70 has changed at least twice. In some species Hsc20’s Hsp70 partner interacts only with the scaffold, in others it has many JDP partners in addition to Hsc20 and interacts with many client proteins. Analysis of this switching of Hsp70 partners has provided insight into the insulation of JDP/Hsp70 systems from one another that can occur when more than one Hsp70 is present in a cellular compartment, as well as how competition among JDPs is balanced when an Hsp70 partner is shared amongst a number of JDPs. Of particularly broad relevance, even though the scaffold’s interactions with Hsc20 and Hsp70 are functionally critical for the biogenesis of FeS cluster-containing proteins, it is the modulation of the Hsc20-Hsp70 interaction *per se* that allows Hsc20 to function with such different Hsp70 partners.

## Introduction

**FIGURE 1:**
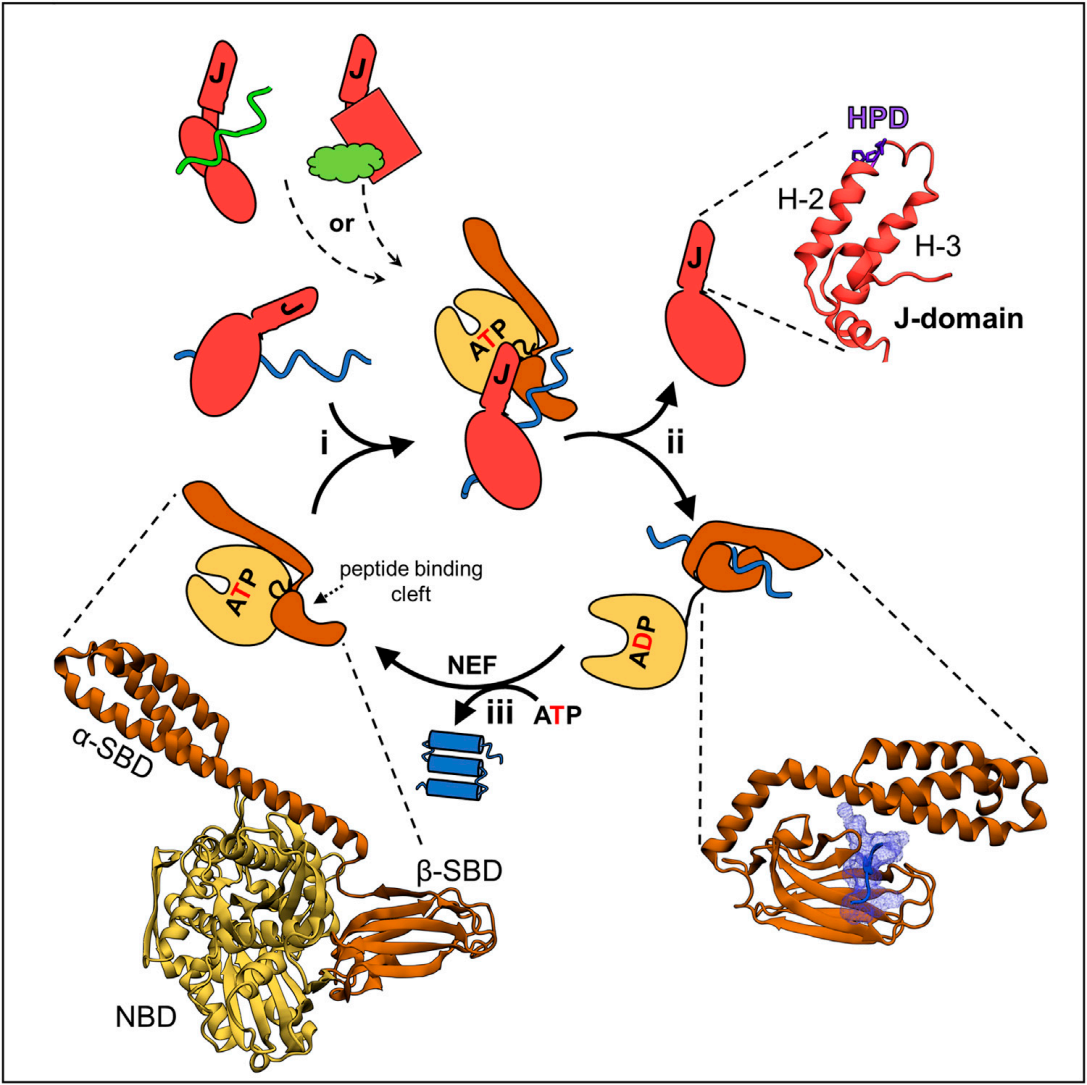
JDP/Hsp70 client binding cycle and structures of key components. (upper left) Different JDPs (red) can deliver different clients, indicated by different colors and shapes, to the same Hsp70 by interacting with them directly *via* distinct client binding domains, indicated by different shapes. (center) This scheme depicts the cycle involved in folding of a client protein. The same cycle occurs when JDP/Hsp70 systems are involved in other cellular processes, including FeS cluster biogenesis (see Figure 3): (i) JDP-client complex interacts with ATP bound Hsp70. In this “open” conformation the nucleotide binding domain (NBD, beige) is docked on to the substrate binding domain (SBD, brown) and the interdomain flexible linker (black) is bound in a crevice of the NBD. This conformation allows easy access of client to the peptide binding cleft of β-SBD, as α-SBD is retracted from it, and enables binding of the J-domain, which interacts at the NBD-β-SBD interface. It also allows simultaneous, transient interactions of the J-domain and JDP-bound client, and thus synergistic stimulation of ATP hydrolysis by Hsp70. (ii) ATP hydrolysis triggers conformational changes resulting in separation of the NBD and SBD, only connected by a flexible linker. The α-SBD subdomain covers the peptide binding cleft of β-SBD, stabilizing its interaction with the client. Separation of the SBD and NBD also results in loss of the J-domain binding face, and thus JDP release. (iii) ADP exchange for ATP, often involving a nucleotide exchange factor (NEF), reverting Hsp70 to the ATP bound state, causing client release. Structures of critical components of the cycle from *E. coli* DnaK and DnaJ—the best studied JDP/Hsp70 system—are depicted (bottom left) the ATP bound conformation of DnaK (PDB ID: 4B9Q). (Upper right) J-domain architecture is conserved with helices 2 and 3 (H-2, H-3) forming a finger like structure. Loop between these helices contains His Pro Asp (HPD) sequence present in all J-domains and required for the stimulation of Hsp70s’ ATPase. (bottom right) Hsp70’s SBD interacting with a peptide derived from a client protein (PDB ID: 1DKZ). Note helical α-SBD subdomain covering the binding cleft of β-SBD, but without contacting the client.

*Via* their cyclic interaction with client proteins, Hsp70-based molecular chaperone systems are key players, not only in general protein homeostasis, but also in central cellular pathways (Clerico et al., 2019; Mayer and Gierasch, 2019; Rosenzweig et al., 2019; Balchin et al., 2020; Liu et al., 2020). This review is focused on such an Hsp70-dependent pathway—the biogenesis of iron-sulfur (FeS) clusters, cofactors necessary for activities of many proteins (Py and Barras, 2015; Maio and Rouault, 2020; Braymer et al., 2021; Esquilin-Lebron et al., 2021). In this pathway, which was inherited by eukaryotic cells from the bacterial progenitor of mitochondria, clusters are assembled on the client scaffold protein, called IscU/Isu in bacteria/mitochondria, and then transferred to recipient proteins. However, regardless of the exact client or cellular function, the Hsp70-client interaction cycle depends on the same conformational changes of Hsp70, which are triggered by binding and hydrolysis of ATP (Figure 1). ATP hydrolysis profoundly affects Hsp70’s affinity for client—resulting in a non-equilibrium enhancement of client affinity (often called ultra-affinity) by minimizing escape of the interacting client (Barducci and De Los Rios, 2015). Client release is driven by subsequent ADP dissociation. This cycle is driven by cochaperones. *Via* action of its J-domain, a J-domain protein (JDP) cochaperone activates the ATPase of Hsp70; ADP release is typically induced by a nucleotide exchange factor (NEF) cochaperone (Mayer and Gierasch, 2019; Liu et al., 2020).

JDPs are key to the functional diversity of Hsp70 systems, in good part by influencing what clients Hsp70 binds (Kampinga and Craig, 2010; Craig and Marszalek, 2017; Zhang et al., 2022). As the name implies, membership in this diverse family of proteins requires only a J-domain. All J-domains, regardless of their role in the cell or their partner Hsp70, have a His, Pro, Asp tripeptide (HPD) in a loop connecting two helices (Figure 1). The HPD plays a critical role in stimulating ATPase activity, while the two flanking helices form the extended Hsp70 binding face. Other JDP domains often bind client polypeptides. These are directly targeted to Hsp70 *via* interaction of the JDP’s J-domain, which then stabilizes the client-Hsp70 interaction by stimulating ATPase activity. J-domains act synergistically with clients, as robust ATPase stimulation occurs only when client interacts in Hsp70’s peptide binding cleft. The JDP involved in the conserved FeS cluster biogenesis system (Hsc20) is highly specialized. Its only client is the Isu/IscU scaffold on which the FeS clusters are built (Vickery and Cupp-Vickery, 2007; Dutkiewicz and Nowak, 2018).

Unlike JDPs, all Hsp70s are structurally similar—having an N-terminal nucleotide binding domain (NBD) and a C-terminal substrate binding domain (SBD) composed of α and β subdomains. β-SBD contains the hydrophobic peptide binding cleft formed by two four-stranded β-sheets and two pairs of upward loops. It binds an extended polypeptide segment of the client—five residues enriched in hydrophobic amino acids (Clerico et al., 2015; Mayer and Gierasch, 2019; Liu et al., 2020; Clerico et al., 2021). α-SBD acts as a lid covering the peptide binding cleft, thus stabilizing client interaction (Figure 1). Initiation of client binding occurs when Hsp70 is in the ATP-bound conformation—in which both SBD subdomains and the linker that separates the SBD from the NBD are docked on the NBD, leaving the peptide binding cleft very accessible. The HPD and Helices 2 and 3 of the J-domain form a binding interface with Hsp70 sequences in the NBD, β-SBD and the linker.

Typically, JDPs function within networks consisting of a limited number of Hsp70s, sometimes just one, cooperating with a larger number of JDPs, including both those involved in general proteostasis and those involved in a singular cellular pathway (Craig and Marszalek, 2017; Barriot et al., 2020; Piette et al., 2021). Such networks are highly dynamic, as the number of JDPs and Hsp70s constituting them, as well their partnerships, vary across evolving lineages (Kominek et al., 2013; Powers and Balch, 2013; Rebeaud et al., 2021). Although such changes are observed in both prokaryotic and eukaryotic lineages, an overall trend is apparent: networks are composed of a larger number of JDP/Hsp70 systems in complex multicellular eukaryotes, i.e., more in animals and plants than in bacteria, archaea or unicellular eukaryotes (Powers and Balch, 2013; Rebeaud et al., 2021). Little is known, however, about the molecular mechanisms underlying expansion, contraction and reorganization of these networks.

Most Hsp70s bind many different substrates. However, there are examples of specialized Hsp70s. Hsp70s that partner with JDP Hsc20 in FeS cluster biogenesis in bacteria and a subset of fungi are of this type (Schilke et al., 2006; Vickery and Cupp-Vickery, 2007). Here we focus on the client-JDP/Hsp70 system involved in FeS cluster biogenesis. This system is biologically important—the client is the central component of a crucial biological pathway present in both prokaryotes and eukaryotes. Moreover, across the tree of life, the dedicated JDP of the system sometimes functions with a specialized Hsp70 and sometimes a multifunctional Hsp70. Consequently, it has proven key to understanding how networks composed of one Hsp70 cooperating with a number of JDPs are able to effectively carry out so many functions in the cell.

## The client Isu/IscU, the scaffold for biogenesis of FeS clusters

**FIGURE 2:**
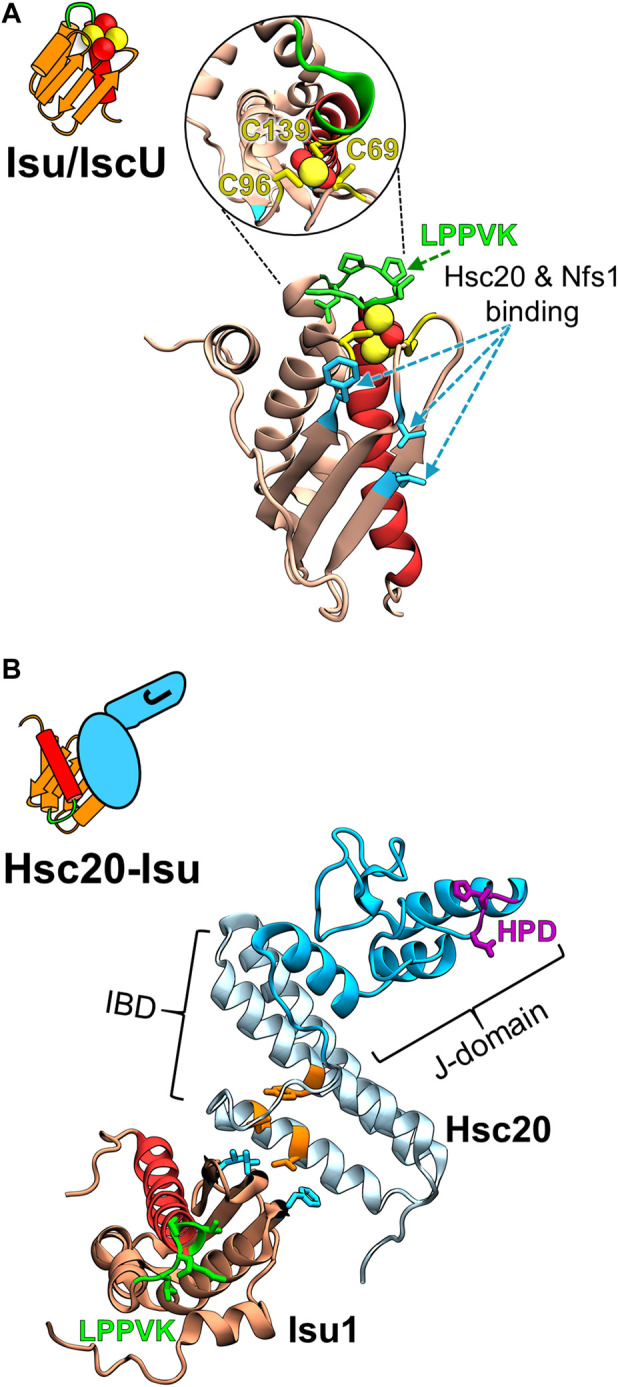
Structural models of the FeS cluster scaffold and its complex with Hsc20. **(A)** Cartoon depicting key structural elements of FeS cluster loaded (yellow/red balls) Isu/IscU scaffold and homology model of *S. cerevisiae* Isu1 with bound FeS cluster based on the crystal structure of FeS cluster bound IscU from *Aquifex aeolicus* (PDB ID: 2Z7E) at left and right, respectively. Insert within the circle is the structure tilted 90° to visualize the three Cys residues (yellow) that coordinate a 2Fe-2S cluster (yellow/red balls). LPPVK sequence (green) localized on a loop prior to C-terminal helix 5 (red) is the binding site for Hsp70. Hot spot residues involved in the Isu1 interactions with both Hsc20 and cysteine desulfurase Nfs1 are indicated (cyan). Note: the Hsc20 and Hsp70 binding sites on Isu1 do not overlap. **(B)** Cartoon representing J-domain protein Hsc20 in complex with Isu/IscU and structural model of the Hsc20 of *S. cerevisiae* (PDB ID: 3UO2 and 3UO3) in complex with Isu1 at left and right, respectively. Hsc20 is a simple JDP consisting of the N-terminal J-domain (dark cyan) with HPD motif (magenta) and C-terminal Isu binding domain, IBD (light cyan), which interacts with Isu *via* a contact surface involving hot spot residues (orange).

The conserved Isu/IscU scaffold protein on which FeS clusters are built is present in the bacteria and in mitochondria of eukaryotes (Maio and Rouault, 2020; Braymer et al., 2021; Esquilin-Lebron et al., 2021). It is a small polypeptide with a compact fold—five α-helices and three β-strands (Kim et al., 2012b; Adrover et al., 2015; Tanaka et al., 2019; Kunichika et al., 2021). It coordinates a 2Fe-2S cluster utilizing three invariant Cys residues and one or more additional residue(s) whose identity is not yet clear (Figure 2). Nuclear Magnetic Resonance (NMR) analyses of bacterial IscU revealed that the scaffold is structurally highly dynamic, populating two conformational states—a structured conformation that resembles the conformation when in complex with an FeS cluster and a disordered conformation (Kim et al., 2012b; Kim et al., 2012c).

**FIGURE 3:**
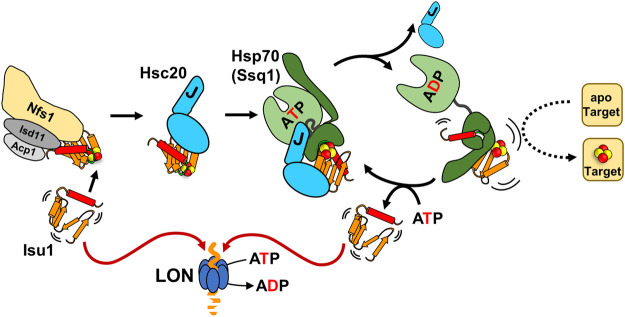
The role of Hsc20/Hsp70 scaffold binding cycle in FeS cluster biogenesis. Interaction of the Isu/IscU scaffold, which is structurally highly dynamic (indicated by agitrons) with a FeS cluster assembly complex (Nfs1/Isd11/Acp1 and other accessory proteins: frataxin, ferredoxin-not shown) is required for *de novo* cluster synthesis. The cluster loaded scaffold interacts with the dedicated JDP Hsc20. The scaffold interactions with both the cluster assembly complex and Hsc20 protect it from degradation by LON protease. Hsc20-scaffold complex interacts with its Hsp70 partner in the ATP bound conformation. Synergistic stimulation of Hsp70’s ATPase by the J-domain of Hsc20 and the scaffold triggers conformational changes that leads to Hsc20 release and stabilization of an Hsp70-scaffold complex. Formation of this complex is required for effective cluster transfer onto apo-target proteins. Exchange of ADP for ATP results in scaffold release. It is not clear what regulates whether a scaffold is reused in another round of cluster assembly/transfer or proteolyzed.

During the process of assembly of the cluster and its subsequent transfer onto target proteins, Isu/IscU interacts with several proteins (Figure 3) (Bonomi et al., 2011; Boniecki et al., 2017; Gervason et al., 2019; Lin et al., 2020). Hsc20 and the cysteine desulfurase which donates sulfur for cluster assembly have overlapping binding sites on a face of the structured Isu/IscU conformation (Figure 2) (Shi et al., 2010; Majewska et al., 2013). These interfaces are conserved across bacterial and eukaryotic systems (Füzéry et al., 2011; Ciesielski et al., 2012; Boniecki et al., 2017; Fox et al., 2019). Hsp70 binds an invariant LPPVK pentapeptide in the flexible loop that is just prior to the C-terminal α-helix (Hoff et al., 2002; Hoff et al., 2003; Dutkiewicz et al., 2004; Bonomi et al., 2011). This short loop has been reported to also be involved in binding of frataxin, a key component of the FeS synthesis complex assembled around cysteine desulfurase (Prischi et al., 2010; Manicki et al., 2014; Fox et al., 2019). Moreover, Isu/IscU is a substrate for the ATP dependent protease LON (known as Pim1 in *Saccharomyces cerevisiae*) (Figure 3) (Ciesielski et al., 2016; Ciesielski and Craig, 2017). Consistent with its structural instability, Isu of *S. cerevisiae* has a LON/Pim1-dependent fast turnover rate *in vivo* (Andrew et al., 2008; Song et al., 2012). Whereas the physiological importance of the high turnover rate is not yet clear, evidence indicates that the high levels of Isu observed under stress conditions are beneficial for the cell. How such regulation may occur is not known.

## Importance of Hsc20 and Hsp70 interaction with the Isu/IscU scaffold for FeS cluster biogenesis

Strong evidence exists for the importance of the binding of Hsc20 and its partner Hsp70 to Isu for functioning of the FeS cluster biogenesis pathway (Figure 3). Studies of *S. cerevisiae*—which unlike bacteria, has no alternative, Hsc20/Hsp70-independent FeS cluster biogenesis pathway (Py and Barras, 2015; Esquilin-Lebron et al., 2021)—demonstrated that the Hsc20-Isu interaction is critical *in vivo* (Ciesielski et al., 2012). Cells are inviable when the binding face of either Hsc20 or Isu is severely compromised (Majewska et al., 2013). When the interaction is partially defective, cells grow slowly and have reduced activities of enzymes that required FeS clusters for catalysis (i.e., succinate dehydrogenase and aconitase). Like the Hsc20-Isu interaction, Isu binding by Hsp70 is critical (Dutkiewicz et al., 2004). *S. cerevisiae* cells expressing variants having either amino acid substitutions in the LPPVK of Isu or the peptide binding cleft of Hsp70 have a “null phenotype” (Dutkiewicz et al., 2004; Knieszner et al., 2005). Partial inhibition of the interaction results in slow growth and reduced activity of enzymes dependent on an FeS cluster for function (Dutkiewicz et al., 2004; Knieszner et al., 2005). That the Isu-Hsc20 interaction is important for delivery of Isu to Hsp70 by the typical client-JDP-Hsp70 interaction cycle is supported by *in vitro* data (Ciesielski et al., 2012; Dutkiewicz et al., 2017). As simultaneous interaction of a J-domain and client are required for robust ATPase stimulation, client-JDP complex formation would be expected to increase the efficiency of stimulation as it increases the chance of both being near Hsp70 at the same time. Consistent with this idea, when the Isu-Hsc20 interaction is compromised, Hsp70’s ATPase activity is stimulated only weakly (Ciesielski et al., 2012). Furthermore, consistent with the typical requirement for ATP hydrolysis for stable Hsp70-client binding, stable Hsp70-Isu complex formation is very inefficient when the Isu-Hsc20 interaction is compromised (Dutkiewicz et al., 2017).

That interactions of Hsc20 and partner Hsp70 with Isu/IscU are important for the transfer of cluster from Isu/IscU onto recipient protein(s) is supported by results of both *in vivo* and *in vitro* experiments. Depletion of either Hsc20 or Hsp70 Ssq1 in *S. cerevisiae* results in increased amounts of FeS cluster bound Isu and reduced activity of FeS cluster requiring enzymes (Mühlenhoff et al., 2003). Similar results were obtained when Hsc20 and Hsp70 Ssq1 variants defective in interactions were tested (Dutkiewicz et al., 2006). The most extensive *in vitro* experiments have been carried out using the bacterial system—transferring cluster from *Escherichia coli* IscU onto recipient apo-proteins including apo-ferredoxin (Fdx) and apo-glutaredoxin 5 (Grx5) (Chandramouli and Johnson, 2006; Bonomi et al., 2008, 2011; Iametti et al., 2015). Transfer did occur in absence of Hsc20 (HscB) and partner Hsp70 (HscA), but at a rate considered lower than expected. The presence of both HscB and HscA accelerated the rate. This acceleration was dependent on ATP hydrolysis, as expected for chaperones using the canonical client binding cycle. It has been hypothesized that Hsp70 binding facilitates cluster release by inducing major conformational change of IscU (Bonomi et al., 2011)—consistent with NMR results demonstrating that HscA binding shifts the IscU population into the disordered conformation (Kim et al., 2012a). But how Hsp70 binding to the LPPVK sequence mechanistically translates into accelerated FeS cluster transfer from Isu onto recipient protein(s) is not yet clear, as structural information is lacking. However, it is not difficult to imagine, given how Hsp70s bind substrates, that Isu would undergo a conformational change that would destabilize the cluster from Isu/IscU—especially considering that the Hsp70 binding site lies in a loop just prior to the C-terminal α-helix that contains a cysteine required for cluster coordination (Bonomi et al., 2011).

## Hsc20 functions with three different Hsp70 partners across the tree of life

**FIGURE 4:**
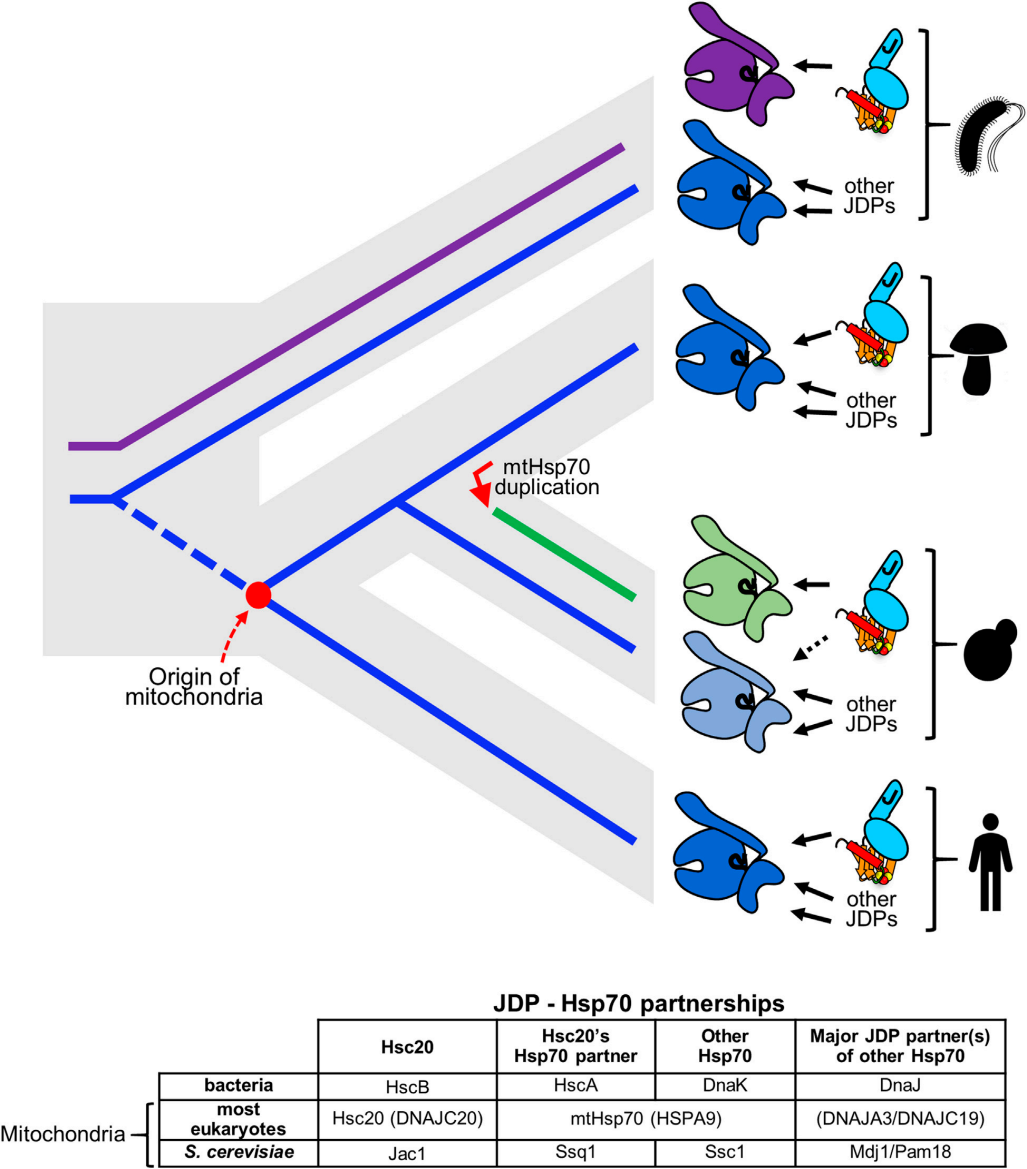
Hsc20 has changed Hsp70 partners twice across the tree of life. (top) Gray contour indicates phylogenetic relationships among evolving lineages: bacteria, fungi, metazoa. Solid lines indicate evolution of Hsp70s HscA (magenta) and DnaK (blue); broken blue line indicates the eukaryotic lineage prior to the origin of mitochondria (red dot). JDP Hsc20 is found in bacteria and mitochondria of eukaryotes (cyan, shown bound to client Isu/IscU in brown with FeS cluster). Solid arrows indicate its Hsp70 partner. In bacteria Hsc20 (HscB) partners with FeS cluster specialized Hsp70 HscA (magenta). This system coexists with multifunctional Hsp70 DnaK (dark blue). In mitochondria of eukaryotic cells Hsc20 partners with mtHsp70 e.g., most fungi and metazoans (indicated by mushroom and human pictograms) or with FeS cluster specialized Hsp70 Ssq1 (green), which evolved after an Hsp70 gene duplication (red arrow) in a progenitor of the *S. cerevisiae* lineage (budding yeast pictogram). The other duplicate, Ssc1 (light blue) remained multifunctional partnering with multiple JDPs, but only minimally with Hsc20, as indicated by dotted line. Following the origin of mitochondria Hsc20 was maintained, but HscA was not. Its function in FeS cluster biogenesis was substituted by mtHsp70, a descendant of DnaK (dark blue). (bottom) Names of JDPs and Hsp70s functioning in FeS cluster biogenesis. Those in parentheses are the name of human proteins.

Although the overall client interaction cycle utilized by Hsc20 and its Hsp70 partner in the FeS cluster biogenesis pathway is typical, it is unusual in that Hsc20 partners with different Hsp70s across evolving lineages (Figure 4). Two of the Hsp70 partners are specialized, interacting with only one client—Isu/IscU; one is multifunctional, interacting with many clients, including Isu/IscU (Pukszta et al., 2010; Delewski et al., 2016; Kleczewska et al., 2020). In bacteria, Hsc20 partners with the specialized Hsp70, HscA (Hesterkamp and Bukau, 1998; Silberg et al., 1998). In *S. cerevisiae* and closely related species Hsc20 functions with a specialized mitochondrial Hsp70, Ssq1 (Schilke et al., 1999; Schilke et al., 2006). However, in mitochondria of most eukaryotes there is only one Hsp70 (mtHsp70). Hsc20 and all other JDPs partner with mtHsp70 (Srivastava et al., 2019; Maio and Rouault, 2020), which is a descendent of DnaK, the multifunctional Hsp70 of bacteria, not HscA (Huynen et al., 2001; Kleczewska et al., 2020). Like DnaK, it binds a wide array of clients, including Isu (Schilke et al., 2006; Srivastava et al., 2019).

Why/how did such shifts in Hsc20/Hsp70 partnership occur? As HscA orthologues have not been found in eukaryotic genomes (Kleczewska et al., 2020), most likely loss of an Hsp70 drove the change to Hsc20 functioning with multifunctional mtHsp70. This shift could have occurred during or before the emergence of mitochondria. HscA, but not Hsc20, may have been lost during the massive transfer of genes from the bacterial endosymbiont to the host cell (Roger et al., 2017; Sloan et al., 2018). Alternatively, mitochondria may have evolved from a bacterium that already lacked HscA, as two bacterial genomes have been found to harbor HscB, but not HscA (Barriot et al., 2020). While the timing of the switch to multifunctional Hsp70 in mitochondria remains elusive, it is clear that the specialist Hsp70 Ssq1 arose during fungal evolution from a duplication of the gene encoding mtHsp70 (Schilke et al., 2006; Kominek et al., 2013; Kleczewska et al., 2020). Thus, though Ssq1 resembles bacterial HscA in its specialization, evolutionarily Ssq1 is not directly related to HscA. Following this duplication, which took place in a common ancestor of *S. cerevisiae* and *Candida albicans*, Ssq1 evolved toward being an Isu-binding, FeS cluster biogenesis specialist. Its paralog, termed Ssc1, maintained the multifunctionality typical of mtHsp70s, binding many clients and its ATPase being activated by several different JDPs (Figure 4).

That either a specialized or a multifunctional Hsp70 can partner effectively with Hsc20 in the FeS cluster biogenesis pathway is not only interesting in its own right, it also provides tools to address some fundamental questions regarding the functioning of JDP/Hsp70 networks in general. Two are discussed in the next sections. First, is the question of insulation of JDP/Hsp70 systems. When more than one Hsp70 is present in a cellular compartment, sometimes JDP/Hsp70 systems function independently from one another. What is the basis of such insulation? And, more fundamentally, how is any network fine-tuned to allow many different JDPs to effectively function with a single Hsp70? Second, what determines the substrate specificity of a specialized Hsp70? How does such specificity arise?

## Hsc20 J-domain interaction with Hsp70 is responsible for functional insulation of specialized systems

Even though they share the same cellular space, the specialized and multifunctional JDP/Hsp70 systems of *E. coli* and the mitochondria of *S. cerevisiae* are functionally insulated from one another. The insulation is more extreme in *E. coli*. Specialized Hsc20 HscB does not function at all with multifunctional DnaK, consistent with the fact that HscB does not stimulate the ATPase activity of DnaK. Nor can DnaJ, the main JDP that functions with DnaK, partner with HscA, even though it can weakly stimulate its ATPase activity (Silberg et al., 1998). Insulation of the systems in mitochondria is not as complete. Hsc20 can work, albeit poorly, with multifunctional Hsp70 Ssc1 (Kim et al., 2001; Pukszta et al., 2010). Although Hsc20 is essential (Voisine et al., 2001), Ssq1 is not (Schilke et al., 1996; Knight et al., 1998). Cells lacking Ssq1 grow very slowly, and even this poor growth requires the upregulated expression of Isu that occurs upon deletion of Ssq1 (Andrew et al., 2008; Song et al., 2012; Dong et al., 2017). However, the reverse is not the case. Ssq1 does not work with other mitochondrial JDPs—Ssq1’s ATPase is stimulated only by Hsc20, not by other mitochondrial JDPs (D'Silva et al., 2003; Dutkiewicz et al., 2003).

**FIGURE 5:**
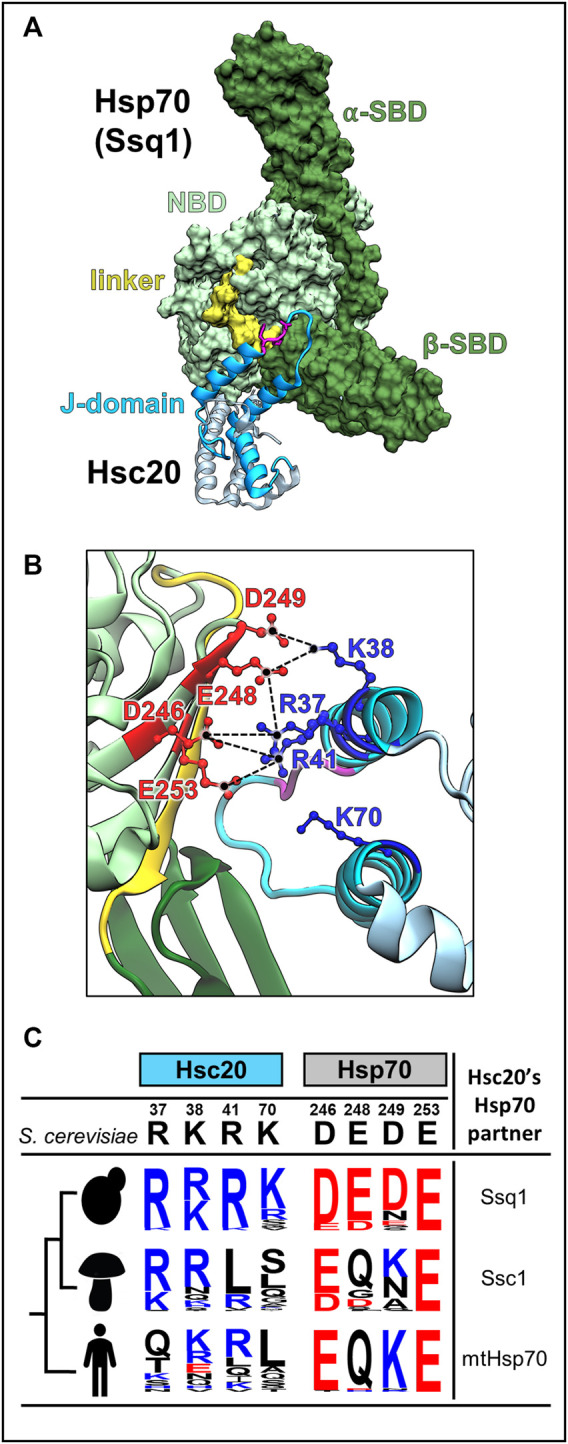
J-domain-Hsp70 interaction involves evolutionary variable interface. **(A)** Structural model of the *S. cerevisiae* Hsc20-Ssq1 (ATP) complex (Tomiczek et al., 2020). As in all JDP-Hsp70 interactions, the J-domain binds at the interface of NBD, β-SBD and interdomain linker formed when Ssq1 is in the ATP-bound conformation. Hsc20 in cyan with J-domain dark, Isu binding domain, light; HPD motif (magenta). Ssq1: NBD (light green), α- and β-SBDs (dark green); interdomain linker (yellow) is bound to the NBD. **(B)** The interconnected network of contacts between positively charged residues of helix 2 of the J-domain (blue) and negatively charged residues of Ssq1 (red) is critical for J-domain interaction with Ssq1; electrostatic interactions are depicted as broken lines (black). Note that one residue on the J-domain side interacts with more than one residue on the Hsp70 side e.g., J-domain’s R41 contacts D246 and E253 of Ssq1. K70 of helix 3 is also critical for Hsc20-Ssq1 complex formation, though its binding partner(s) was not identified. **(C)** The key residues involved in the J-domain-Ssq1 interaction are evolutionary variable. Sequence logos represent the amino acid frequency at positions of Hsc20 and Hsp70, which are homologous to the hot spot positions of the *S. cerevisiae* Hsc20 J-domain-Ssq1 interface; positively charged (blue), negatively charged (red) and uncharged (black). The analysis was based on Hsc20 and Hsp70 sequences from 23 yeast species, which diverged after Hsp70 gene duplication leading to Ssq1, 47 pre-duplication fungi species (harboring only mtHsp70) and 20 metazoan species (harboring mtHsp70), designated by human figure. Note: in contrast to the invariant HPD motif hot spot residues involved in J-domain/Ssq1 interaction in *S. cerevisiae* are evolutionary variable, particularly outside the post-duplication species.

Recent data suggests that the key to this functional insulation of JDP/Hsp70 systems specialized in FeS cluster biogenesis is the J-domian-Hsp70(ATP) interaction (Pukszta et al., 2010; Delewski et al., 2016). Overall, the mode of J-domain interaction across systems—*via* the HPD motif and helices 2 and 3—is conserved (Figure 5A) (Kityk et al., 2018; Wang et al., 2021). However, while the HPD is invariant, the residues of helix 2 and 3 forming the Hsp70 binding face are not conserved. Critical interfacial positions are occupied by very different amino acids, even in relatively closely related species (Figures 5B,C) (Delewski et al., 2016; Tomiczek et al., 2020). Perhaps most importantly, these interfaces are networks of interconnecting residues—key residues on one side of an interface form contacts with more than one residue on the other side (Figure 5B). Such contact networks are known to be highly flexible (Keskin et al., 2008; Aakre et al., 2015). During the course of evolution contacts can be added, deleted or replaced, as long as enough contacts are maintained across the interface that interaction remains (Levin et al., 2009). They enable coevolution between the interacting partners—change(s) on one side of the interface can be compensated by a complementary change(s) on the other (Szurmant and Weigt, 2018; Cong et al., 2019).

Such changes could explain how new partnerships between JDPs and Hsp70s have formed and how functionally insulated JDP/Hsp70 systems have emerged. For example, the emergence of the insulated Hsc20/Ssq1 system could have occurred in the following way: Immediately following the gene duplication, sister copies of mtHsp70 were identical, and Hsc20 was able to interact and function with both. However, *via* J-domain/Hsp70 coevolution the affinity of Hsc20 for one paralogue (Ssq1) increased such that a new, more specific, partnership was formed. This new partnership was advantageous as it allowed Hsc20 to avoid competing with other JDPs for Hsp70 access. Thus, strengthening of this partnership was likely driven by positive selection, accelerating the coevolutionary changes. Furthermore, a gain of high specificity toward the LPPVK by Ssq1, as discussed in the next section, made reversion to multifunctionality unlikely (Pukszta et al., 2010; Delewski et al., 2016; Tomiczek et al., 2020).

The existence of the insulated JDP/Hsp70 systems in bacteria and mitochondria raises the question of whether other functionally insulated JDP/Hsp70 systems exist. The best documented example is the specialized Hsp70 (Ssb) in the cytosol of *S. cerevisiae* (James et al., 1997) (Huang et al., 2005; Zhang et al., 2017). Ssb arose *via* gene duplication from the multifunctional Hsp70 (Ssa) during the emergence of fungi. It functions with a specialized JDP termed Zuo1. In metazoans, including humans, the orthologue of Zuo1 functions with the multifunctional Hsp70, which is closely related to Ssa—thus, resembling the situation of mitochondrial Hsc20/mtHsp70 system (Hundley et al., 2005). Considering the high copy number dynamics observed for both Hsp70s and JDPs across the tree of life, it is likely that other functionally insulated JDP/Hsp70 systems await discovery (Barriot et al., 2020; Rebeaud et al., 2021; Zhang et al., 2022).

## Specialization of Hsp70 client binding arose from restricting its ability to bind multiple clients

HscA and DnaK bind the LPPVK peptide derived from IscU with similar affinities. The mode of binding by the two Hsp70s is similar and fundamentally resembles that of β-SBDs with other client peptides—in an extended conformation in the peptide binding cleft with the α-helical lid (α-SBD) covering it without directly interacting (Cupp-Vickery et al., 2004; Clerico et al., 2015; Clerico et al., 2021) (Figure 1). However, while most peptides bind Hsp70 with the central position of the cleft occupied by a Leu, Val, or Phe, in the case of LPPVK it is occupied by the second Pro residue (Cupp-Vickery et al., 2004). Furthermore, LPPVK binds in the reverse orientation from that of most peptides—C to N backbone direction (Tapley et al., 2005; Tapley et al., 2006), rather than the more typical N to C (Clerico et al., 2021).

HscA, however, does not bind the peptide NRLLLTG that is most commonly used in studies of DnaK and other multifunctional Hsp70s (Zhu et al., 1996). There are only a small number of amino acid differences between HscA and DnaK β-SBDs in or near the peptide binding cleft (Tapley et al., 2006; Vickery and Cupp-Vickery, 2007). DnaK-like substitutions at these positions in HscA allow interaction with the NRLLLTG peptide, suggesting that the client specificity of HscA evolved by remodeling the peptide binding cleft, not by changing the mechanism of peptide binding *per se*. Although no structural data is available for the Ssq1 and Ssc1 Hsp70 pair, their SBDs, like those of HscA and DnaK, bind the LPPVK peptide with very similar affinities. Furthermore, the SBDs of Ssq1 do not bind other peptides recognized by Ssc1 (Schilke et al., 2006). Thus, like HscA, Ssq1 likely evolved high specificity toward LPPVK by restricting its ability to accommodate sequences other than LPPVK and provides an explanation for why this increased specificity is not accompanied by an increased affinity.

Considering that multifunctional mtHsp70 binds LPPVK and other pentapeptide substrates with similar affinity it is possible that the system could tolerate changes in LPPVK without disrupting function. Why then has this sequence remains invariant across all species, even those engaging multifunctional mtHsp70 in FeS cluster biogenesis. A possible explanation is provided by biochemical experiments demonstrating the direct involvement of LPPVK in Isu’s interaction with frataxin (Manicki et al., 2014). Since frataxin is a key player in FeS cluster biogenesis in all eukaryotes, evolutionary conservation of LPPVK may have been driven by its dual function (Srour et al., 2020; Braymer et al., 2021; Esquilin-Lebron et al., 2021).

## Additional roles of Hsc20-Isu binding beyond Hsp70 cochaperone activity in FeS cluster transfer

Though Hsc20 is a simple, small JDP, consisting only of a J-domain and an Isu binding domain (Cupp-Vickery and Vickery, 2000; Bitto et al., 2008; Ciesielski et al., 2012), the results of both *in vivo* and *in vitro* experiments have raised the possibility that Hsc20 binding to Isu/IscU has effects in addition to targeting it to Hsp70 for binding. These results are discussed below as they raise the intriguing question of whether JDPs can evolve to be intimately intertwined in cellular pathways, and more generally how the diverse JDP family of proteins evolves.

Hsc20 binding affects both Isu/IscU’s structural stability and its susceptibility to LON protease. HscB binding promotes the shift of IscU towards the structured conformation (Kim et al., 2012a) and binding of HscB to cluster loaded IscU in the absence of HscA reduces the rate of cluster transfer below that measured for IscU alone (Iametti et al., 2015). Overproduction of Hsc20 in *S. cerevisiae* leads to elevated levels of Isu (Ciesielski et al., 2016). This *in vivo* effect on Isu levels may go beyond structural stabilization; it may directly affect recognition of Isu by LON (Ciesielski and Craig, 2017). Hydrophobic residues serve as LON recognition sequences (Pinti et al., 2016). Replacement of hydrophobic residues in the Hsc20 binding site of Isu with serines slows down degradation of Isu by LON *in vitro* (Ciesielski and Craig, 2017).

The overlap of Hsc20 and desulfurase binding sites on Isu/IscU, and thus mutual exclusivity of binding, places Hsc20 at the center of the FeS cluster biogenesis pathway—at the transition from cluster assembly to transfer (Figure 3) (Majewska et al., 2013). It is tempting to speculate that the Hsc20/Hsp70 system plays roles in controlling both physiological levels of cluster loaded Isu and flow of FeS clusters through the biogenesis pathway. Such regulatory roles would likely involve Hsc20-dependent displacement of cluster-loaded Isu from the complex with cysteine desulfurase and facilitation of FeS cluster transfer onto recipient proteins *via* delivery of cluster loaded Isu to Hsp70. But they could also involve regulating the levels of Isu. Not surprisingly, since the Isu binding site of cysteine desulfurase that provides the sulfur for the cluster assembly overlaps that of Hsc20 (Majewska et al., 2013), desulfurase also protects Isu from LON degradation (Andrew et al., 2008; Song et al., 2012; Ciesielski et al., 2016).

In thinking about the possible roles of Hsc20 in the complex FeS cluster biogenesis pathway, it is potentially informative to consider how they may have evolved. The original Hsc20 progenitor may have had no J-domain or connection to Hsp70s at all. Its role may have been to control FeS cluster biogenesis potential, that is IscU levels by reversibly interacting and protecting it against proteolytic degradation. However, this protective function may have been in adaptive conflict with cluster transfer, as stronger interaction with IscU would have been good for protection but bad for transfer and *vice versa*. This adaptive conflict could have been resolved by recruitment of a J-domain because interaction with Hsp70 facilitated release of IscU from the complex, thus lifting its inhibitory effect on the cluster transfer. Moreover, Hsp70 interaction with IscU further accelerated the cluster transfer rate. Similar scenarios could explain evolution of other specialized JDPs involved in other cellular pathways, e.g., splicing (Sahi et al., 2010), histone biogenesis (Hammond et al., 2021; Piette et al., 2021) and gene silencing (Ichino et al., 2021).

## Conclusion

Studies on the specialized Isu/IscU-Hsc20/Hsp70 FeS cluster biogenesis systems have provided insights into previously underappreciated aspects of client-chaperone interactions that are broadly applicable to JDP/Hsp70 systems. First, the plasticity of the J-domain-Hsp70 interaction allows modulation of the JDP-Hsp70 interactions and thereby drives the ability of one Hsp70 to productively function with several JDPs, as well as the insulation of networks when more than one Hsp70 is present. Second, some JDPs may be more involved in the underlying biological pathway in which they are involved than generally recognized—having evolved by either gaining a J-domain or by gaining other interactions after already being an active JDP cochaperone for Hsp70.
